# The Extracellular Superoxide Dismutase Sod5 From *Fusarium oxysporum* Is Localized in Response to External Stimuli and Contributes to Fungal Pathogenicity

**DOI:** 10.3389/fpls.2021.608861

**Published:** 2021-03-02

**Authors:** Qiang Wang, Ambika Pokhrel, Jeffrey J. Coleman

**Affiliations:** Department of Entomology and Plant Pathology, Auburn University, Auburn, AL, United States

**Keywords:** cell wall protein, cotton, *Fusarium* wilt, glycosylphosphatidylinositol anchor, reactive oxygen species, superoxide dismutase, virulence factor

## Abstract

Reactive oxygen species (ROS) produced by hosts serve as a general defense mechanism against various pathogens. At the interaction site between the host and pathogen, host cells rapidly accumulate high concentrations of ROS, called the oxidative burst, that damage and kill the invading microbes. However, successful pathogens usually survive in a high ROS environment and have evolved strategies to overcome these detrimental effects. Here we characterized the biological function of the extracellular superoxide dismutase (SOD) FoSod5 from *Fusarium oxysporum* f. sp. *vasinfectum*. *FoSOD5* is strongly up-regulated during infection of cotton, and a Δ*FoSOD5* mutant was significantly reduced in virulence on cotton. Purified 6 × His-FoSod5 could significantly inhibit the reduction of NBT and WST-1, indicating that FoSod5 was a functional SOD protein. Based on CRISPR/Cas9 technology, several different FoSod5 variants were generated and used to assess the secretion, expression, and subcellular localization of FoSod5 in *F. oxysporum*. The subcellular localization of FoSod5 is altered under different environmental conditions. During normal growth conditions, FoSod5 was primarily localized to the phialides; however, in a nutrient-limited environment, FoSod5 was localized to a wide array of fungal structures including the septum and cell wall. FoSod5 is an alkaline-induced glycosylphosphatidylinositol (GPI) protein and the GPI anchor was required for proper protein subcellular localization. The multiple mechanisms fungi utilize to tolerate the oxidative burst is indicative of the importance of this plant defense response; however, the presence of a conserved extracellular SOD in many phytopathogenic fungi suggests tolerance to ROS is initiated prior to the ROS entering the fungal cell.

## Introduction

The ascomycete fungus *Fusarium oxysporum* is an important pathogen that can infect and cause disease on a wide range of hosts including plants, animals, and humans (Gordon, 2017). Over 100 formae speciales have been described within the *F. oxysporum* species complex based on their ability to cause disease on different host plants (Michielse and Rep, 2009). *F. oxysporum* f. sp. *vasinfectum* (*Fov*) is responsible for *Fusarium* wilt of cotton, and is a significant disease found worldwide in all growing areas (Davis et al., 2006). This soil-borne fungus invades the vascular tissue via the roots and rapidly spreads to the aboveground portion of the host. Typical field symptoms of *Fusarium* wilt include yellowing, chlorotic leaves, dark-brown/necrotic xylem, and wilting, eventually leading to plant death (Davis et al., 2006).

Reactive oxygen species (ROS) are produced by host cells and play an important role in defense against various pathogens. These small ROS molecules are highly toxic to the infective agents and are able to directly eliminate them. At the interaction site between the host and pathogen, plant cells rapidly synthesize and accumulate large quantities of ROS via a membrane-bound NADPH oxidase during a process called the oxidative burst (Lamb and Dixon, 1997; Heller and Tudzynski, 2011). In plants, the oxidative burst may result in the hypersensitive response (HR) which inhibits the spread of the pathogen to surrounding tissue (Zurbriggen et al., 2010), and can serve as an important signal that initiates a series of other plant defense responses or the production of plant hormones (Sauer et al., 2001). These processes stimulate host plants to alter the expression of genes involved in defense response, leading to the production of phytoalexins, callose deposition, and systemic acquired resistance; thereby impeding further pathogen spread and disease development (Sauer et al., 2001; Forman et al., 2010).

Despite the extensive production of ROS during the plant defense response, successful pathogens are usually able to survive in an environment with a high ROS concentration and have evolved mechanisms to overcome the detrimental effects of this defense response (Aguirre et al., 2006; Fones and Preston, 2012). The family of enzymes known as superoxide dismutases (SOD) participates in catalyzing the partitioning of superoxide radicals. SOD enzymes are divided into several classes based on the structure of the enzymes and the specific binding of a metal cofactor, and include the copper and zinc SODs (Cu/Zn SOD), the iron or manganese SODs (Fe/Mn SOD), and the nickel SODs (Miller, 2012). The cytosolic Cu/Zn SODs are the most common SOD enzymes in eukaryotic cells and there is at least one gene encoding a Cu/Zn SOD in a fungal genome (Miller, 2012). In *Saccharomyces cerevisiae*, loss of *SOD1* results in slow growth and increased sensitivity to H_2_O_2_ and ROS-generating compounds (Moradas-Ferreira and Costa, 2000). Additionally, the loss of function of *SOD1* can alter normal fungal physiology. For instance, a *SOD1* mutant of the ericoid mycorrhizal fungus *Oidiodendron maius* has reduced production of conidia and the capacity for mycorrhization (Abba et al., 2009).

Extracellular SODs have recently been described in various pathogens (Robinett et al., 2018). In fungi, extracellular SODs typically contain an N-terminal secretion signal peptide and a C-terminal glycosylphosphatidylinositol (GPI) anchor attachment site. The mature GPI anchor enables the protein to be localized to the cell membrane and/or cell wall by covalent attachment (Mayor and Riezman, 2004; Robinett et al., 2018). Some fungal extracellular SODs are secreted out of the cell and participate in catalyzing the partitioning of superoxide radicals produced during a host defense response enabling survival in a high ROS environment. The genome of *Candida albicans* encodes a Cu-only SOD (Sod5) which contributes to pathogen tolerance to ROS (Gleason et al., 2014). The *C. albicans* Sod5 rapidly binds and sequesters a copper co-factor from the host, compromising copper toxicity to *C. albicans* (Li et al., 2015a). Similarly, an extracellular SOD in the dimorphic fungus *Histoplasma capsulatum* contributes to resistance to host-derived oxidative stress in yeast cells (Youseff et al., 2012). In *Puccinia striiformis*, the causative agent of stripe rust on wheat, a secreted extracellular Zn-only SOD contributes to enhanced resistance to oxidative stress during the interaction between the wheat host and the fungus (Liu et al., 2016). Collectively, these studies indicate extracellular SODs play an important role in pathogenicity on various hosts and confer tolerance to the host derived oxidative stress caused by ROS.

Extracellular SODs are predicted to have evolved from the canonical *SOD1* and are phylogenetically conserved throughout fungi (Robinett et al., 2018), and therefore are postulated to share a similar biological function. In this study, an extracellular SOD (FoSod5) from *Fov* was important for tolerance to multiple ROS and was required for full virulence on cotton. The subcellular localization of FoSod5 was dependent on the environmental condition, where FoSod5 was primarily localized to the fungal phialides, but when exposed to a high ROS environment the enzyme was localized to the septum and cell wall. This demonstrates that some phytopathogenic fungi utilize extracellular SODs to tolerate the oxidative burst during the plant defense response.

## Materials and Methods

### *Fusarium oxysporum* Isolate and Growth Conditions

The race 7 *Fov* isolate was obtained from the Fungal Genetics Stock Center (FGSC #10442) (McCluskey et al., 2010), and the wild-type, mutants, and complemented strains (listed in Supplementary Table 1) were grown and maintained at room temperature (25°C) on Potato Dextrose agar (PDA; Difco, Franklin Lakes, NJ, United States) medium or minimum nutrient medium (M-100) (Stevens, 1974).

Fungal sensitivity to various chemical stressors was evaluated by placing a 5 mm diameter mycelial plug from the edge of a 4-day-old culture grown on M-100 agar medium and put onto the new agar medium containing the chemical. The diameter of the colony was evaluated after 7 days. Each treatment was repeated three times.

### Bioinformatic Analysis

The SOD protein family in *F. oxysporum* was identified by BLASTp against the EnsembleFungi database^1^ using several well characterized SOD proteins as query sequences (*S. cerevisiae* SOD1: YJR104C and SOD2: YHR008C; *C. albicans* SOD5: XP_719507). Protein domains were predicted using PFAM (El-Gebali et al., 2019) and BLASTp software. The subcellular localization peptide sequences were identified with SeqNLS and WoLF PSORT (Horton et al., 2007; Lin and Hu, 2013), and secreted signal peptides were predicted with SignalP 4.1 (Petersen et al., 2011). Multiple sequence alignments were conducted with T-Coffee (Notredame et al., 2000), and a phylogenetic tree was generated with PhyML 3.1 (Guindon et al., 2010). The 3D protein structure of FoSod5 was predicted using the SWISS-MODEL server (Gleason et al., 2014; Waterhouse et al., 2018).

### Gene Disruption and Complementation of *FoSOD5*

The target gene, *FoSOD5*, was disrupted with the hygromycin B resistance cassette using a previously described split-marker approach with a few modifications (Goswami, 2012). Generation of protoplasts was conducted as previously described (Coleman et al., 2011; Wang et al., 2018) using a mixture of 10 mg/mL Driselase^TM^ (Sigma, St. Louis, MO, United States), 15 mg/mL β-Glucanase (Sigma), and 5 mg/mL Lyzing enzymes (Sigma) for 2-3 h. DNA fragments were generated by overlapping fusion PCR with the oligomer pairs, olFoSOD51F/olFoSOD51R, HYGF1/HYGR2, olFoSOD53F/olFoSOD54R, and HYGBF3/HYGBR4 (Goswami, 2012). Transformants were selected on 150 μg/mL hygromycin B, and colonies appearing after 3–5 days were transferred to M-100 medium with hygromycin B (150 μg/mL) for further evaluation.

An *Agrobacterium*-mediated transformation method was used to generate the *FoSOD5* gene complementation strain as described in a previous publication with a few modifications (Mullins Chen et al., 2001). The *FoSOD5* gene complementation plasmid was constructed using pCAMBIA1302 (Cambia, Canberra, Australia) as the background plasmid where the 35S promoter and hygromycin phosphatase gene located between the *Xho*I and *Kpn*I restriction sites was replaced with the neomycin phosphotransferase II cassette (trpC promoter) amplified from the pII99 plasmid (Namiki et al., 2001) using primers NeoF_*Kpn*I and NeoR_*Xho*I. An approximately 4 kb locus containing the *FoSOD5* gene was amplified from *Fov* genomic DNA using the primers FoSOD5cassF and FoSOD5cassR, and ligated into the above constructed plasmid between the LB- and RB-T-DNA sequences using the NEBuilder^®^ HiFi DNA Assembly Cloning Kit (New England Biolabs, Ipswich, MA, United States), generating the desired complementation plasmid, pCom-G418^R^-*FoSOD5*.

The Δ*FoSOD5* strain was co-cultivated with the *A. tumefaciens* strain *AGL-1* with plasmid pCom-G418^R^-*FoSOD5* and placed in the dark at 28°C for 2 days as described (Mullins Chen et al., 2001). Selection of the desired complemented strain was accomplished by plating on M-100 medium containing 60 μg/mL G418 and 300 μg/mL cefotaxime, and grown at room temperature for 5–7 days. G418 resistant transformants were isolated to new selective medium for screening by PCR and Southern blot. The Southern blot was conducted according to the manufacturer’s instructions (DIG-High Prime DNA Labeling and Detection Starter Kit I, Roche). PCR DIG probes for detecting the hygromycin cassette and *FoSOD5* cassette were generated with the primer pairs HyBF/HyBR and olFoSOD55F/olFoSOD56R, respectively. The *Sal*I restriction enzyme was used to confirm the hygromycin cassette copy number, while the *Bam*HI restriction enzyme was used to detect FoSOD5 cassette copy number. All the primers used in this study are listed in Supplementary Table 2.

### 6 × His-FoSod5 Protein Purification and SOD Activity Assay

The 6 × His-FoSod5 protein purification was conducted using a previously published method (Gleason et al., 2014). Briefly, the middle sequence of *FoSOD5* encoding the SOD domain (lacking the regions encoding the N-terminus signal peptide and the C-terminus GPI-anchor site) was inserted into the pHis-Parallel1 vector (Sheffield et al., 1999), and the recombinant plasmid was transformed into *Escherichia coli* Rosetta^TM^(DE3) competent cells. Protein production was induced with the addition of 0.5 mM IPTG to a culture with an OD_600_ value between 0.5 and 0.6 and allowed to grow for 4 h at 37°C. Protein purification was performed under denaturing conditions with prepared denaturing buffer (50 mM Tris–HCl (pH 8.0), 8 M urea, and 1.5 mM reduced glutathione) using a Ni-NTA Purification System (Thermo Fisher Scientific, Waltham, MA, United States). A series of dialysis buffers containing a decreasing gradient of concentration of urea (50 mM Tris–HCl (pH 8.0), 6/4/2/0 M urea, and 0.25 mM oxidized glutathione) were sequentially used for FoSod5 protein refolding with a 10 kDa dialysis tube. SOD proteins usually require different metals to fold into the correct structure for function, and different metal ions (20mM of ZnCl_2_, FeCl_3_, CuSO_4_, and MnSO_4_) were provided individually to the 6 × His-FoSod5 during the final overnight dialysis. The final 6 × His-FoSod5 protein was concentrated to 2 mg/mL with a 3 K protein concentrator tube (MilliporeSigma, Burlington, MA, United States).

Two methods, the nitro-blue tetrazolium (NBT) method and the SOD-WST assay, were used for assessing the enzymatic activity of the purified 6 × His-FoSod5. The NBT method reflects SOD enzyme efficiency through inhibition of the reduction of NBT when superoxide is present. When NBT is reduced by superoxide, formazan is produced resulting in a proportional dark blue color change that can be monitored. Three mL of the freshly prepared reaction buffer (10 μM riboflavin, 45 μM EDTA, 350 μM NBT, 60 μM methionine, and potassium phosphate with a pH value of 7.8) was added to different concentrations (0, 4, 10, 20, and 40 μg) of the purified 6 × His-FoSod5 protein, mixed, and immediately incubated under light (∼4,000 Lx) for 20 min at 28°C (Durak et al., 1993). Presented data is a representative set of samples based on three independent experiments.

The SOD-WST assay was conducted using the EnzyChrom^TM^ SOD Assay Kit (BioAssay Systems, Hayward, CA, United States) according to the manufacturer introductions. One μg of the FoSod5 protein that was reconstituted in the presence of the different metal cations was added to the WST-1 reaction buffer and incubated at room temperature for 1 h. The result was detected by measuring the absorbance at 440 nm (Cytation 3, BioTek, Winooski, VT, United States). Two independent experiments were conducted and the average and standard deviation presented.

### Pathogenicity Assay

For the cotton root infection assay, cotton seeds (cultivar FM1944) were planted in sterile soil and placed in a growth chamber with a 16 h 28°C light/8 h 24°C dark cycle. After 2 weeks, cotton plants at the two-to-three true leaf stage were used in the infection assay. The cotton seedlings were carefully uprooted and immersed in sterile water to remove adhering soil from the root system. A set of eight seedlings were transplanted to a water-culturing box containing nutrient solution (1/10 Murashige and Skoog medium (MS, PhytoTechnology Laboratories, Shawnee Mission, KS, United States) supplemented with 0.1% sucrose) to sustain the growth of the cotton plant and allowed to acclimate for 4 days in the growth chamber.

The wild-type and mutant strains of *Fov* inoculum were prepared by growing on a rotary shaker in 100 mL PDB at room temperature for 5 days. The conidia were collected from the cultures, washed in sterile water, and suspended at a final concentration of 1 × 10^7^ conidia/mL. Two mL of the conidia were pipetted into the water-culturing box containing the cotton plants and mixed well with gentle shaking. Each week the nutrient solution in the plant culturing box was replaced with fresh nutrient solution. After 5–8 weeks, disease symptoms were recorded by photo and the vascular tissue of the cotton plant was evaluated. Based on disease symptoms of *F. oxysporum* on cotton, four disease index categories were defined: 0 – no symptoms, 1 – light yellowing of the leaves and black roots present, 2 – heavy chlorosis of the leaves, wilting evident, and brownish discolored vascular tissue, 3 – wilted plant with dark discolored vascular tissue or death of the plant. Pathogenicity assays for each strain were conducted at least twice.

### RNA Extraction of *Fov*-Infected Cotton Roots and qRT-PCR

Preparation and inoculation of the cotton plants (FM1944) was slightly different than the pathogenicity assay. Instead of geminating seeds in soil, they were germinated in 1/2 strength MS medium polymerized with 2.6 g/L of phytagel. After 1 week of germination, the plants were transferred to a water-culturing box containing 1/2 strength MS nutrient solution. When the plants were 2 weeks old, the MS medium in the box was replaced with 1/10 MS medium supplemented with 0.1% sucrose and inoculated with ∼2 × 10^7^ conidia in each box. The three randomly selected *Fov*-infected cotton roots from three plants were pooled for each time point and samples were collected at multiple time-points (0, 4, 12, 24, and 36 h, 2, 3, 5, and 7 days post inoculation) for total RNA extraction. Total RNA was extracted using the E.Z.N.A.^®^ Total RNA Kit I (Omega Bio-Tek, Norcross, GA, United States) according to the manufacturer instructions, and the total RNA was treated with DNase I (New England Biolabs). Reverse transcription was immediately conducted to generate cDNA using the QuantiTect Reverse Transcription Kit (Qiagen). Transcript expression of *FoSOD5* was determined by qRT-PCR on a Bio-Rad CFX96 instrument for the different time points of fungal infection using primers qFoSOD5F and qFoSOD5R (Supplementary Table 2) and the *F. oxysporum* elongation factor 1 alpha gene (*EF1*α) was used as the internal reference gene using primers qEF1αF and qEF1αR (Supplementary Table 2). The relative expression levels were calculated using the ΔΔ*Ct* method (Livak and Schmittgen, 2001). Two biological replicates (each with three technical replicates) were performed in this experiment.

### Plasmid Construction for Generation of GFP and LacZ Reporter FoSod5 Strains

The HITI Cas9 RNP transformation plasmid, pUC19-HITI-*FoSOD5*C2, was constructed using the NEBuilder^®^ HiFi DNA Assembly Cloning Kit and four PCR fragments were simultaneously assembled into the pUC19 plasmid between the *Bam*HI and *Hin*dIII restriction sites. The first fragment contained a ∼1 kb upstream sequence and partial amino acid coding region (AA: 1–53; primers: NA_FoSod5C1hitiF and NA_FoSod5C2uphitiR); the second fragment was the *sGFP* coding sequence (primers: NA_FoSod5C2midsGFPF and NA_FoSod5C2midsGFPF); the third fragment contained a partial amino acid coding region (AA: 186-end containing the GPI site) and the predicted native terminator of the *FoSOD5* gene (∼800 bp) (primers: NA_FoSod5C2downF and NA_FoSod5C2downR); and the forth fragment was the hygromycin cassette amplified from plasmid pUC19-HDRI-FoSSO1-mCherry using primers: NA_FoSod5C2HYGBF and Universal_NA_forHITITR. The plasmid pUC19-HITI-*FoSOD5*C2 contained most of the *FoSOD5* gene with the exception that the catalytic SOD domain was replaced by *sGFP*. The plasmids, pUC19-HITI-*FoSOD5*C1 and pUC19-HITI-*FoSOD5*C3 were generated in a similar method. Plasmid pUC19-HITI-*FoSOD5*C1 lacked all the amino acid sequences after the SOD domain, while in pUC19-HITI-*FoSOD*5C3 the entire ORF of *FoSOD5* gene was replaced by the β*-galactosidase* (*lacZ*) coding region cloned from plasmid, pCYC-lacZ (gift from Dr. Paul Cobine, Auburn University). The three different plasmids were used to transform the WT *Fov* isolate to generate the FoSod5 variants enabling the study of the FoSod5 function including protein secretion, protein subcellular localization, and protein expression (Wang and Coleman, 2019). All PCR primers used are listed in Supplementary Table 2.

### *In vitro* Cas9 Nuclease Assay and Cas9 RNP Transformation

Before Cas9 RNP transformation, an *in vitro* Cas9 nuclease assay was conducted to confirm the Cas9 RNP cleavage activity (Wang et al., 2018). The Cas9 RNP mediated transformation was conducted as previously described (Wang and Coleman, 2019), and the amount of donor plasmid did not exceed 6 μg per transformation plate. After transformation, positive colonies were isolated on M-100 selective medium containing 150 μg/mL hygromycin. Three pairs of PCR primers were used to confirm the location of the integrated plasmids. Since four different FoSod5 variants were generated using the HITI strategy, the cleavage site at the 5′-terminus (SS, Supplementary Figure 3) was sequenced.

### β-Galactosidase (LacZ) Activity Assay

The expression and regulation of *FoSOD5* was investigated under various conditions. For different pH values, M-100 agar medium with a final X-gal concentration of 200 ng/mL was adjusted with a pH ranging from 6 to 8. *FoSOD5* expression under the influence of different carbon sources was investigated using M-100 medium as a base where the glucose component was replaced with 1% (w/v) of various carbon sources (glycerol, carboxymethylcellulose (CMC), starch, sucrose, sorbitol, and mannitol). ∼1 × 10^5^ conidia were dropped onto the center of the agar plates with or without the X-gal. For quantification of β*-Galactosidase* enzymatic activity, an O-Nitrophenyl-B -D-Galactopyranoside (ONPG) assay was conducted according to two previous protocols (Pardee et al., 1959; Smale, 2010). The *proFoSod5:LacZ* strain was cultured to the exponential stage in YG liquid medium. Different chemicals (0.05% H_2_O_2_, 0.5 mM diamide, 0.2 mM menadione, 20 μg/mL xanthine oxidase/0.1 mM hypoxanthomine, and 150 μM CuSO_4_) were added to the YG medium and the fungi were cultured for an additional 3 h. Total protein was extracted with liquid nitrogen, and 5 μg of total protein (in 75 μL) from each treatment and 40 μL of 4 mg/mL ONPG were added to 700 μL reaction buffer (8.5 mg/mL Na_2_HPO_4_, 5.5 mg/mL Na_2_H_2_PO_4_**.**H_2_O, 750 ng/mL KCl, and 246 ng/mL MgSO_4_**.**7H_2_O, pH 7.0) and incubated at 30°C for 25 min. 100 μL of 1M Na_2_CO_3_ was used to stop the above reaction, and the β*-Galactosidase* enzymatic activity was measured by absorbance at 420 nm (Cytation 3, BioTek, Winooski, VT, United States). All assays were repeated in triplicate.

### Secreted Protein Extraction and Western Blotting

A trichloroacetic acid (TCA) precipitation method was used to extract the fungal secreted proteins. The fungal isolates were cultured in YG liquid medium for 3 days at room temperature. The resulting fungal culture was centrifuged at 13,000 *g* for 10 min and filtered through a 0.22 μM filter to remove hyphae and conidia. TCA was added to a final concentration of 10%, and the mixture chilled at 4°C overnight. The solution was centrifuged at 13,000 *g* for 10 min, and the resulting pellets were washed with ice-cold acetone at least twice. The final protein pellets were dissolved in 50 mM Tris–HCl buffer (pH = 7.5), and the protein concentration was determined using a Qubit 3.0 Fluorometer with the Qubit^TM^ Protein Assay Kit (Thermo Fisher Scientific).

Western blotting was conducted with a total of 5 μg of the secreted proteins which were separated by 10% SDS-PAGE gel electrophoresis and stained with Coomassie staining solution (0.1% Coomassie Brilliant Blue R-250, 50% methanol, and 10% glacial acetic acid). The proteins in the SDS-PAGE gel were transferred to a nitrocellulose filter membrane (GE Healthcare, Chicago, IL) using the Mini Trans-Blot^®^ Electrophoretic Transfer Cell (Bio-Rad, Hercules, CA, United States). Anti-GFP antibody (Rockland Immunochemicals, Gilbertsville, PA, United States) and an ECL chemiluminescent detection kit (GE Healthcare) were used to detect the resulting proteins.

### Confocal Microscopy

Confocal microscopy was conducted with a Nikon A1 Confocal Microscope with an excitation wavelength of 488 nm for detection of sGFP fluorescence as previously described (Wang and Coleman, 2019). For different chemical treatments, a mixture of hyphae and conidia was inoculated into YG medium and cultured for 3 days. One mL of the hyphae-conidial mixture was transferred into fresh YG medium and cultured on a rotary shaker at 18°C, 220 rpm for 16 h. Assayed chemicals (0.03% H_2_O_2_ and 20 μg/mL xanthine oxidase/0.1 mM hypoxanthomine) were added to the YG medium and cultured for an additional 2 h. After this time, samples were aliquoted, fixed with 4% paraformaldehyde, and observed by confocal microscopy using an inverted agar method (Hickey and Read, 2009). All microscopy experiments were repeated at least three independent times.

## Results

### Identification of an Extracellular GPI Anchored SOD Protein (FoSod5) From *F. oxysporum*

**FIGURE 1:**
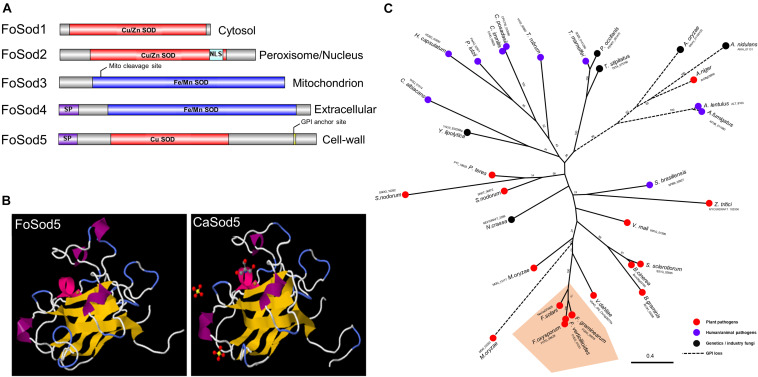
Domain, structure, and phylogenic analysis of FoSod5. **(A)** Identification of the Sod protein family in *F. oxysporum* and predicted subcellular localization of FoSod proteins. NLS: nuclear localization sequence; SP: secreted peptide. **(B)** The predicted protein structure of FoSod5 and comparison with CaSod5. **(C)** The maximum-likelihood phylogenetic tree of Sod5 from ascomycete fungi. The tree was generated with a bootstrap value of 500.

Several Sod proteins from various fungal species were used as a query and identified five SOD proteins in the *F. oxysporum* genome. These SOD encoding genes included three Cu/Zn SOD genes (*FoSOD1*: FOTG_01421; *FoSOD2*: FOTG_16882; *FoSOD5*: FOTG_08628) and two Fe/Mn SOD genes (*FoSOD3*: FOTG_10379; *FoSOD4*: FOTG_02058) (Figure 1A). Prediction of the subcellular localization indicated FoSod1 (a Cu/Zn SOD) was localized to the fungal cytosol while FoSod3 (a Fe/Mn SOD) was localized to the mitochondrion, in agreement with the subcellular localization of homologous SOD proteins from other fungi (Luk et al., 2003; Yao et al., 2016). However, FoSod2 (a Cu/Zn SOD) was predicted to be localized to either the peroxisome and/or the nucleus while FoSod4 (a Fe/Mn SOD) is predicted to have a secretion signal peptide and could be a secreted SOD enzyme. FoSod5, contains an N-terminal secretion signal (AA: 1–21) and a C-terminal glycophosphatidyl inositol (GPI) attachment site (AA: 240), suggesting this protein is secreted and anchored to the fungal cell wall or membrane (Figure 1A). Sequence alignment of selected Cu/Zn SODs revealed that FoSod5 lacks two histidine residues involving in zinc binding implicating FoSod5 is a Cu-specific SOD enzyme (Supplementary Figure 1; Gleason et al., 2014). Although the amino acid sequence similarity between CaSod5 and FoSod5 was only 33%, the predicted structure of FoSod5 was highly similar to CaSod5, suggesting functional similarities between the two proteins (Figure 1B). Phylogenetic analysis indicated many fungi in the Ascomycota contain a single ortholog of *SOD5* (Figure 1C), and isolates within *Fusarium* carried a single phylogenetically conserved copy of the *SOD5* gene (Figure 1C). Interestingly, all the genomes from the *Aspergillus* species included in this analysis appear to have lost the GPI anchor site during their evolution, but it had been retained in the closely related *Penicillium* spp. indicating the loss of the GPI anchor occurred after the divergence between these two genera (Figure 1C).

### FoSod5 Is a Functional SOD Protein

**FIGURE 2:**
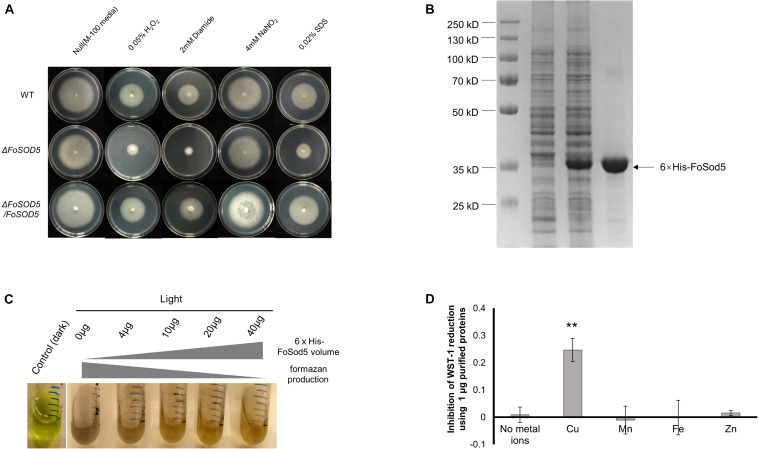
FoSod5 is a functional SOD protein and confers tolerance to ROS. **(A)** Evaluation of fungal sensitivity to different chemical stressors. The indicated strains were inoculated on minimal medium (M-100). The assay was repeated three times. **(B)** SDS-PAGE gel depicting protein expression and purification conditions. Lane 1 represents total protein before inducing; Lane 2 total protein after addition of 0.5 mM IPTG for 4 h; Lane 3 represents purified 6 × His-FoSod5 protein. **(C)** Assessment of purified 6 × His-FoSod5 enzymatic activity using the nitro-blue tetrazolium (NBT) method. The production of formazan decreases as the 6 × His-FoSod5 protein increases. Three independent replicates per treatment were conducted. **(D)** Determination of metal ion co-factor preference for 6 × His-FoSod5 using the SOD-WST assay. Only copper ions were able to confer SOD activity. The average inhibition and standard deviation is depicted in the histogram and is based on two independent experiments.

A mutant of *FoSOD5* was generated to assess the contribution of the SOD to oxidative stress tolerance (Supplementary Figure 2). The Δ*FoSOD5* mutant had increased sensitivity to hydrogen peroxide and diamide, a ROS-generating compound, in a radial growth ROS stress assay (Figure 2A). Functional SOD activity for FoSod5 was confirmed using two *in vitro* methods, the NBT method and the SOD-WST1 assay. The middle amino acid sequence harboring the SOD domain was fused with a N-terminal 6 × His tag for protein purification (Figure 2B). This soluble purified 6 × His-FoSod5 protein was used for functional assessment of SOD enzymatic activity and determination of the specificity for different metal ions to serve as a cofactor.

The traditional NBT-riboflavin method indicated the 6 × His-FoSod5 protein was a functional SOD enzyme. Under illumination, the riboflavin-methionine mixture produces superoxide causing NBT to be reduced to the blue colored formazan, and this reaction can be inhibited by SOD activity. A concentration series of the 6 × His-FoSod5 protein (0 to 40 μg) were added to the reaction buffer, and as the concentration of the 6 × His-FoSod5 protein increased, a decrease in the production of formazan was evident (Figure 2C), indicating that FoSod5 is a SOD enzyme able to inhibit the reduction of NBT. Using the SOD-WST1 assay to determine heavy metal cofactor specificity, of all the heavy metals tested only copper was able to confer SOD activity, demonstrating that FoSod5 was a copper-specific SOD (Figure 2D), and is consistent with previous findings on the SOD5 class of enzymes (Gleason et al., 2014).

### *FoSOD5* Is Up-Regulated During Infection and Is Required for Full Virulence of *Fov* on Cotton

**FIGURE 3:**
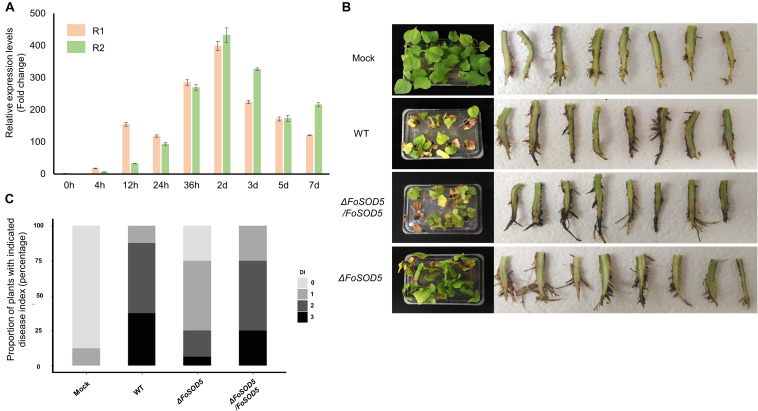
*FoSOD5* is up-regulated *in planta* and involved in virulence on cotton. **(A)** The expression of *FoSOD5* during infection as quantified by qRT-PCR using cDNA from infected plant tissue. *FoSOD5* transcripts were significantly increased 24 h post inoculation. The R1 and R2 data sets represent two independent experiments with three technical replicates. **(B)** Virulence assay depicting cotton symptoms caused by an infection of WT, Δ*FoSOD5*, and Δ*FoSOD5/FoSOD5* strains and control group (Mock). Whole plant symptoms are shown on the left side of the panel, and necrotic development on the root on the right side of the panel. Two independent experiments were conducted. **(C)** The disease index on cotton for the WT, Δ*FoSOD5*, and Δ*FoSOD5/FoSOD5* strains. Ratings were scored from 0 to 3, where 0 was a healthy plant with no symptoms and three indicated a wilted plant with discolored vascular tissue or plant death.

Many fungal virulence factors have increased expression during infection of host plants. As SOD enzymes are known to be involved in virulence in other fungal pathogens (Heller and Tudzynski, 2011), the expression profile of *FoSOD5* during the infection process of cotton was investigated by qRT-PCR. The expression of *FoSOD5* was relatively low during the initial 12 h of infection, but the gene was significantly increased in expression from 24 h to 7 day after infection with the peak being at 2 days (Figure 3A). From 24 h after infection, the average increase in the number of *FoSOD5* transcripts was at least >100-fold (Figure 3A).

As *Fov* is responsible for wilting symptoms, a virulence assay was conducted with the WT, *FoSOD5* mutant (Δ*FoSOD5*), and complemented (Δ*FoSOD5/FoSOD5*) strains on whole cotton plants to assess the role of *FoSOD5* in infection. While the wild-type isolate was able to colonize the xylem tissue in the roots and cause necrosis, the Δ*FoSOD5* strain exhibited reduced necrosis (Figures 3B,C). Overall, the cotton plants inoculated with the *FoSOD5* mutant displayed less wilting symptoms, had less browning of the leaves, and there was less necrosis of the root xylem when compared to cotton plants inoculated with the WT isolate and the Δ*FoSOD5/FoSOD5* complemented strains (Figures 3B,C).

### Expression of *FoSOD5* Is Regulated by the Carbon Source and Is Repressed in a Nutrient Rich-Medium

The function of cell wall proteins can vary and be species-specific, contributing to the diverse properties of the cell wall and enabling fungi to adapt to adverse environments (Gow et al., 2017). In order to investigate the expression of *FoSOD5* in various environmental conditions, a lacZ reporter construct was generated under the control of the native *FoSOD5* promoter (Supplementary Figure 3). The *lacZ* gene, encoding a β-galactosidase, is frequently used as a reporter gene for the *in vivo* analysis of gene regulation in various organisms. After transformation, eight independent colonies were selected for analysis, and seven of these transformants produced the blue pigment when grown on M-100 medium containing X-gal, indicating the expression of *lacZ* is a suitable reporter for the *in vivo* analysis of gene regulation in *F. oxysporum* (Supplementary Figure 4). Sequencing revealed that DNA repair occurred without errors at the sgRNA cleavage site in six of the seven transformants (Supplementary Figure 5, SS site), while the remaining transformant had a 77 bp nucleotide deletion (Supplementary Figure 5).

**FIGURE 4:**
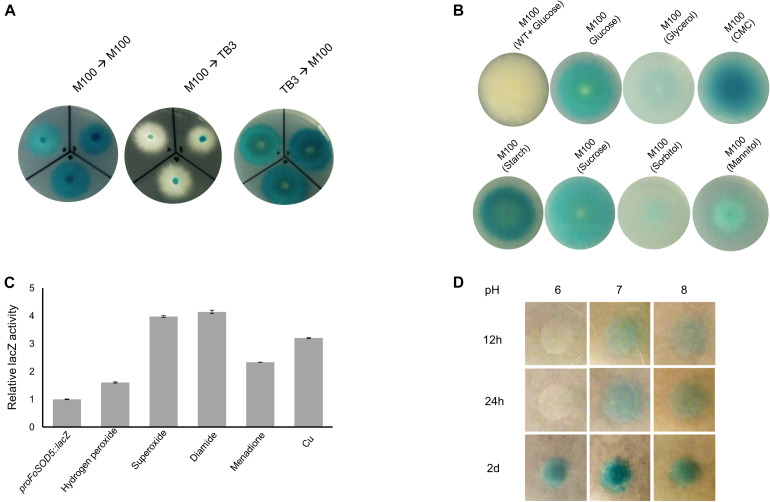
*In vivo* expression analysis of *FoSOD5* under different environmental conditions via the *lacZ* reporter strain. **(A)** Production of β-galactosidase in the *lacZ* reporter strain of *FoSOD5* is dependent on the medium that the fungus is grown. M-100 is a minimal nutrient medium and TB3 is a rich medium. **(B)** The *FoSOD5* lacZ reporter strain produced a varying intensity of blue pigment when grown on M-100 medium supplemented with various sources of carbon (1% w/v). CMC = carboxymethylcellulose. **(C)** β-galactosidase activity of LacZ under different ROS and ROS-generating chemical stimuli. **(D)** The effect of pH on *FoSOD5* gene expression. In a neutral pH or slightly alkaline environment, expression was evident within the first 12 h after treatment. All the assays were replicated three times.

A *proFoSOD5:lacZ* reporter strain was used to investigate the expression pattern of *FoSOD5* when grown in various carbon sources. LacZ activity was evident when the fungus was grown on a minimal nutrient medium (M-100) for 4 days. When an agar plug (5 mm in diameter) was transferred from the *lacZ* inducing M-100 plate to a rich nutrient medium (TB3), the hyphae that grew on the TB3 medium had reduced LacZ activity after 4 days and the hyphae were primarily white in color (Figure 4A). Even after 1 week, there was no significant color change of the mycelia on TB3 media. When an agar plug of the *proFoSOD5:lacZ* reporter mycelia that was grown on TB3 was placed on M-100 medium, the mycelia had LacZ activity. This regulation of LacZ activity by the *FoSOD5* promoter indicates that the expression of the SOD is dependent on the available nutrient(s).

As the carbon sources for the M-100 and TB3 media were different, it was hypothesized that the carbon source in the medium may influence the expression *FoSOD5*. To evaluate this possibility, the 1% (w/v) glucose content in the M-100 agar medium was replaced with 1% (w/v) of various other carbon sources (sucrose, mannitol, glycerol, CMC, starch, and sorbitol). Four days after inoculation, LacZ activity was evident for mycelia grown on all carbon sources investigated, but there was a clear difference between nutrients. Interestingly, the LacZ activity of the mycelia in the presence of CMC and starch were higher than those for the other carbon sources (Figure 4B), suggesting that plant-derived carbon sources may favor *FoSOD5* expression. In addition, *FoSOD5* gene regulation was investigated in the presence of different chemical stimuli including hydrogen peroxide and the ROS generating compounds diamide and menadione. The LacZ activity of the mycelia was increased by these compounds, in particular hypoxanthomine and diamide which had a four fold higher level of LacZ activity than background (Figure 4C).

### *FoSOD5* Is More Rapidly Expressed Under an Alkaline Environment

Many virulence factors of phytopathogenic fungi, including those in *F. oxysporum*, are dependent on the pH of the surrounding environment, and therefore it was hypothesized that the pH of the medium may play a role in the regulation of *FoSOD5*. Conidia of the *proFoSOD5:lacZ* reporter strain were placed on M-100 plates that were adjusted to a pH ranging from 6 to 8 and monitored over time. In a neutral or alkaline environment (pH = 7 or 8), *FoSOD5* was induced within the first 12 h; however, mycelia at a pH 6 did not display LacZ activity until 48 h (Figure 4D).

### The SP and GPI Anchor of FoSod5 Are Required for Subcellular Localization

**FIGURE 5:**
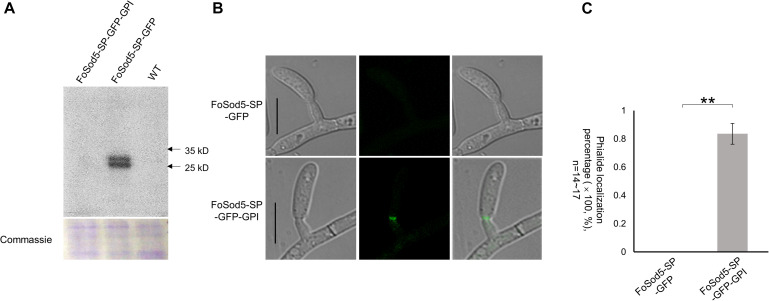
The SP and GPI anchor are required for FoSod5 subcellular localization within *Fov*. **(A)** Western blot analysis with anti-GFP of total protein found in the culture medium of FoSod5 derivatives and the WT. Coomassie protein staining indicated the same amount of loaded protein samples. **(B)** Phialide localization of FoSod5-SP-GFP and FoSod5-SP-GFP-GPI by fluorescence microscopy. **(C)** Statistical analysis of phialide localization among FoSod5-SP-GFP and FoSod5-SP-GFP-GPI, where n represents the number of phialides counted at a given time. Three counts from independent biological samples were used for the statistical analysis. Scale bars represents 10 μm.

Glycosylphosphatidylinositol proteins may contain an N-terminus secretion signal peptide in addition to the C-terminus GPI site (Mayor and Riezman, 2004), which enables attachment to either the fungal cell wall or the plasma membrane (De Groot et al., 2005; Ouyang et al., 2013). Two FoSod5 variants under the control of the native promoter were generated to assess the N-terminal secretion signal peptide of FoSod5 (Supplementary Figure 3b). A homologous-independent targeted integration (HITI) approach was used to insert an entire plasmid at the *FoSOD5* locus (Supplementary Figure 3a), generating a functional *FoSOD5* gene and the desired *FoSOD5* GFP reporter variant in close proximity to one another. The FoSod5-SP-GFP variant included the sequence encoding the first 53 amino acids of FoSod5 containing the signal peptide and *sGFP*, removing the nucleotides encoding the SOD domain and the GPI site (AA: 53 – 263). The second variant, FoSod5-SP-GFP-GPI, replaced the internal catalytic SOD domain (AA: 53 – 186) with sGFP maintaining the GPI anchor attachment site (SP + sGFP + GPI) (Supplementary Figure 3b). After transformation, the region of interest of three FoSod5-SP-GFP and two FoSod5-SP-GFP-GPI transformants were sequenced to confirm they did not contain indels or other undesired alterations (Supplementary Figure 5). These FoSod5 variants and the wild type isolate were grown in liquid culture and after 4 days the supernatant was evaluated for the presence of sGFP by western blot using an anti-GFP antibody. sGFP was only detected in the liquid culture medium from FoSod5-SP-GFP (Figure 5A), confirming FoSod5 contained a functional N-terminal secretion peptide. When the GPI anchor was present (FoSod5-SP-GFP-GPI) the protein was unable to be detected in liquid culture medium, even when ∼25 μg of the extracted protein was used for the western blot and the exposure time for detection was increased.

Comparison of the two sGFP constructs, FoSod5-SP-GFP and FoSod5-SP-GFP-GPI, which only differed with the presence of the GPI site, allows the influence of the GPI anchor on cellular localization to be evaluated. When the FoSod5-SP-GFP-GPI strain was grown in YG medium, a vast majority of the fluorescent signal was observed at the phialides by confocal microscopy while no fluorescence was observed for the FoSod5-SP-GFP variant (Figures 5B,C). This finding, taken together with the previous finding that sGFP was detected by western blotting in the supernatant of the FoSod5-SP-GFP transformant, indicates the GPI site of FoSod5 is required for proper physiological localization at the septum of the phialides when grown in YG medium.

### External Environmental Conditions Influence the Subcellular Localization of FoSod5

**FIGURE 6:**
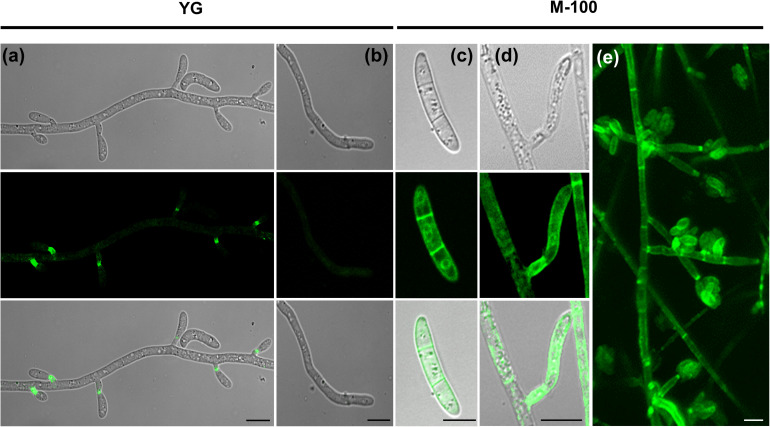
FoSod5-SP-GFP-GPI subcellular localization under confocal microscopy **(a,b)** FoSod5-SP-GFP-GPI subcellular localization in YG medium **(c–e)** FoSod5-SP-GFP-GPI subcellular localization in M-100 medium. **(e)** The *Z*-projection of FoSod5-SP-GFP-GPI on M-100 medium under confocal microscopy. Scale bars represent 10 μm.

FoSod5 was primarily localized to the fungal phialides in YG media (Figures 5B, 6a); however, a weak fluorescent signal could also be observed in conidia, hyphal tips, septum, and hyphae (Figures 6a,b). When this isolate was grown in minimal nutrient M-100 medium, FoSod5 was distributed within an extensive array of fungal structures including the conidia, hyphae, and septa, indicating a significant alteration of the subcellular localization (Figures 6c–e).

Since the FoSod5-SP-GFP-GPI variant did not contain the SOD domain, it was uncertain if this domain had an influence on the subcellular localization of FoSod5. To address this concern, another FoSod5 variant was generated (FoSod5-SP-GFP-SOD-GPI) which contained 2 × *sGFP* in front of the SOD domain (Supplementary Figure 3b). Under confocal microscopy, the FoSod5-SP-GFP-SOD-GPI variant had a similar localization pattern as FoSod5-SP-GFP-GPI indicating the functional SOD domain was not required for proper subcellular localization (Supplementary Figure 6).

## Discussion

Host cells impede invading fungal pathogens through the rapid production of ROS by membrane-bound NADPH oxidases (Marino et al., 2012); and successful fungal pathogens must evolve strategies to overcome the cellular damage from ROS during infection (Heller and Tudzynski, 2011). For instance, *Magnaporthe oryzae* has a robust anti-oxidant defense system conferring high tolerance to the host oxidative burst generated by the host rice plant (Samalova et al., 2014). It has been well established that fungal SOD proteins, in particular members of the Cu/Zn family of SOD proteins, contribute to ROS tolerance by detoxification of reactive superoxide radical anions (Veluchamy et al., 2012; Li et al., 2015b). Collectively, multiple studies have indicated that fungal SOD proteins play an important role as an antioxidant and contribute to fungal pathogenicity.

In fungi, the number of *SOD* encoding genes in a genome varies greatly. The genome of *S. cerevisiae* only encodes two *SOD* genes (*SOD1* and *SOD2*) while some filamentous fungi, including *F. oxysporum*, encode five or more *SOD* encoding genes, eluding to a complex, selective process during the expansion of the *SOD* gene family. Usually expansion of a protein family leads to divergence of protein function. Bioinformatic analysis predicts that the five SOD enzymes of *F. oxysporum* are localized to different organelles or the cytosol. FoSod2 and FoSod5 are predicted to reside in the perioxisome/nucleus and cell wall/membrane, respectively; however, phylogenetic analysis suggests these two SODs may have evolved from the cytosolic FoSod1 (a Cu/Zn SOD). Another example is FoSod4, which is similar to the mitochondrial localized FoSod3, although FoSod4 is predicted to contain a secretion signal peptide at its N-terminus. These results indicate that Sod proteins are translocated to different organelles, which likely influences their physiological function. Increasing evidence has shown that Sod proteins have various subcellular locations and could be localized to alternative sites under stressful conditions. In *S. cerevisiae*, the Cu/Zn SOD enzyme Sod1 is mainly distributed in the cytosol, but rapidly relocates to the nucleus in response to high endogenous and/or exogenous ROS, aiding in the maintenance of genomic stability and serving as a nuclear transcription factor by binding DNA promoter regions of oxidative resistance and repair genes (Tsang et al., 2014).

Most extracellular Sod proteins only use copper as the heavy metal co-factor and function under Zn-limited conditions. A majority of fungi contain 1 to 3 genes encoding extracellular Sod proteins (Broxton and Culotta, 2016), and some of these Cu extracellular Sods have been shown to be involved in fungal pathogenicity in the clinically relevant fungi *C. albicans* and *H. capsulatum*; however, none have been characterized in phytopathogenic fungi. The extracellular Sod FoSod5 from *F. oxysporum* is a virulence factor for cotton, as an *FoSOD5* mutant caused less severe wilt symptoms and had less xylem colonization in infected cotton plants when compared to the wild-type. *FoSOD5* was gradually up-regulated during infection, suggesting FoSod5 may participate in ROS scavenging during the infection process, and mutation of *FoSOD5* led to increased sensitivity to ROS.

While extracellular Sod5-like proteins are widely distributed among most pathogenic fungi and may contribute to virulence during infection, there are exceptions. Deletion of the orthologous GPI-anchored SOD from *Fusarium graminearum*, a close relative to *F. oxysporum* and the main causal agent of *Fusarium* head blight on wheat, failed to have any effect on fungal pathogenicity (Rittenour and Harris, 2013). While the discrepancy between the role of the *SOD5* orthologs in virulence on plant hosts could be due to multiple factors, it is important to note that these fungi infect different host tissues, where *F. oxysporum* is mainly responsible for damage to host roots and colonization of the xylem while *F. graminearum* infects the wheat spikelets, the leaf sheath, and the culm. In addition, *FoSOD5* from *Fov* was gradually upregulated during infection of cotton, indicating a host-induced expression profile; whereas the *SOD5* ortholog from *F. graminearum* did not have a host-induced expression profile and had low expression *in planta* (Yao et al., 2016). Therefore, the regulation of the *SOD5* orthologs is different between these two pathogens, and whether this is due to the regulatory mechanisms at the species level or the *in planta* environment remains unknown.

Expression of *FoSOD5* in *F. oxysporum* was highly dependent on environmental conditions, as in a nutrient-limited (M-100) medium it was highly expressed. In addition, different carbon resources or stresses also influenced the expression of *FoSOD5*, a phenomenon also observed in *C. albicans* when yeast cells were treated with osmotic or oxidative stress conditions (Martchenko et al., 2004). All these results suggest Sod5 plays an important role in fungal adaptation to different environments.

*Fusarium oxysporum* is known to secrete peptides (F-RALF) that induce alkalization in plants and enhance fungal virulence during infection (Masachis et al., 2016; Fernandes et al., 2017), and MAPK-mediated fungal growth on cellophane is more invasive at pH 7 than that at pH 5 (Masachis et al., 2016). Plants inoculated with a *F. oxysporum f-ralf* mutant display ROS accumulation (Masachis et al., 2016), an indication of programmed cell death due to the pathogen. As *FoSOD5* was induced in the presence of high ROS conditions and an alkaline environment, *FoSOD5* could play a critical role in fungal infection.

Previous studies indicated fungal cell wall associated proteins are highly dynamic, dependent on the surrounding environment, and species-specific (De Groot et al., 2005; Klis et al., 2010). The localization of FoSod5 was dependent on the culture environment, where FoSod5 accumulated at the fungal phialides in YG medium. ROS production has been implicated as a signal for fungal differentiation and increases during conidiogenesis (Heller and Tudzynski, 2011). The localization of FoSod5 at the phialides indicates it might act as a ROS scavenging enzyme during conidiogenesis. However, there was no significant difference in the number of conidia produced between the wild-type and *FoSOD5* mutant in YG medium (data not shown), indicating there are likely other factors involved in ROS scavenging during conidiogenesis or FoSod5 has an alternative role at the fungal phialides. In support of additional factors, increased expression of the MnSOD encoding *SOD2* gene in *Colletotrichum graminicola* is evident during generation of conidia (Fang et al., 2002). Additionally, regulation of FoSod5 appears to be more complex in M-100 medium as it was found in multiple locations including the cell wall/membrane, septum, conidia, and phialides. Collectively these results indicates FoSod5 alters its subcellular localization based on environmental cues and might facilitate adaptation to different environments.

The critical role the oxidative burst plays in plant defense has been well established and pathogens have utilized multiple mechanisms to overcome this defense mechanism. Several members of the Cu/Zn Sod family that are predicted to reside in the cytosol serve as virulence factors on plant hosts; however this study indicates that tolerance to ROS begins before the toxic compounds even enter the fungal cell though extracellular SODs. Given the conserved nature of these SODs, this mechanism may potentially serve as a target for development of alternative management strategies for several fungal diseases, including *Fusarium* wilt.

## Data Availability Statement

The original contributions presented in the study are included in the article/Supplementary Material, further inquiries can be directed to the corresponding author/s.

## Author Contributions

QW and JC conceived and designed the experiments and composed the manuscript. QW and AP conducted the experiments. All authors contributed to the article and approved the submitted version.

## Conflict of Interest

The authors declare that the research was conducted in the absence of any commercial or financial relationships that could be construed as a potential conflict of interest.

## Footnotes

1 https://fungi.ensembl.org/index.html
