# Supplementary material for: The Extracellular Superoxide Dismutase Sod5 From Fusarium oxysporum Is Localized in Response to External Stimuli and Contributes to Fungal Pathogenicity
Source: Front Plant Sci. 2021 Mar 2;12:608861. doi: 10.3389/fpls.2021.608861 (PMC7960929; doi:10.3389/fpls.2021.608861)
Supplement: Supplementary file 1 [file Data_Sheet_1.docx]

**Supplemental material**

**The extracellular superoxide dismutase Sod5 from *Fusarium oxysporum* is localized in response to external stimuli and contributes to fungal pathogenicity**

Qiang Wang, Ambika Pokhrel, and Jeffrey J. Coleman

**Table S1.** Strains of *F. oxysporum* used in this study

| Strain | Genotype description |
| --- | --- |
| FGSC 10442 | Wild type (WT) |
| *ΔFoSOD5* | *FoSOD5* gene deletion derived from WT |
| *ΔFoSOD5/FoSOD5* | The complementation strain for *ΔFoSOD5* |
| FoSod5-SP-GPF | EGT^CRISPR^-based transformation of WT expressing SP+sGFP |
| FoSod5-SP-GFP-GPI  FoSod5-SP-GFP-SOD-GPI | EGT^CRISPR^-based transformation of WT expressing SP+sGFP+GPI  EGT^CRISPR^-based transformation of WT expressing SP+sGFP+SOD+GPI |
| pro*FoSOD5*::*lacZ* | EGT^CRISPR^-based transformation of WT expressing *lacZ* under the native *FoSOD5* promoter |

SP: secreted peptide; GPI: glycosylphosphatidylinositol sites; EGT^CRISPR^: CRISPR/Cas9-mediated endogenous gene tagging system.

**Table S2.**  The sequences of oligonucleotides used in this study

| Primer names | Oligomers sequences | Use |
| --- | --- | --- |
| olFoSOD51F | ACGGAGGGTTCCATTCCGTCA | *FoSOD5* gene disruption using a split-marker approach |
| olFoSOD52R | TTGACCTCCACTAGCTCCAGCCAAGCCTGACCACTAGGGCCCGACAGA |  |
| olFoSOD53F | GAATAGAGTAGATGCCGACCGCGGGTTCGGCGGTTAGGGGTAACGGG |  |
| olFoSOD54R | TCCTCGGCTTGGCCTGCATC |  |
| HYGF1 | GGCTTGGCTGGAGCTAGTGGAGGTCAACGGGGATCCTCTAGAGTCGACGG |  |
| HYGR2 | GTATTGACCGATTCCTTGCGGTCCGAA |  |
| HYGF3 | GATGTAGGAGGGCGTGGATATGTCCT |  |
| HYGR4 | AACCCGCGGTCGGCATCTACTCTATTC |  |
| olFoSOD57F | CGGAATCCGGGCTCGGAGAA | Detection of *hph* cassette integration locus in potential *FoSOD5* mutants |
| olFoSOD58R | GCAAGCGTCAGCGGATCTGG |  |
| olFoSOD55F | GCCGCAATCGACGGCAATGT |  |
| olFoSOD56R | GGGAGCGTAGCAGACGAGGC |  |
| H_out_R | GGTCGAGCGTGGTGGCTTGA |  |
| H_out_F | GTCGATGCGACGCAATCGT |  |
| NeoF_KpnI | CGGGGTACCACACCCGAAAAGTATCGACT | *FoSOD5* gene complementation for *FoSOD5* mutants using a *Agrobacterium*-mediated transformation method |
| NeoR_XhoI | CCGCTCGAGTAATGCATTGCAGATGAGCTGTAT |  |
| pCom_FoSOD5R | AGATTGAATCCTGTTGCCGGT |  |
| pCom_FoSOD5F | ACACCCGAAAAGTATCGACTC |  |
| FoSOD5cassF | caagaccggcaacaggattcaatctCTGCTCGCTGGTCGATAG |  |
| FoSOD5cassR | gccggagtcgatacttttcgggtgtGATTTGGGGGCAGAAGTG |  |
| HyBF | TAAATAGCTGCGCCGATGGT | Southern blot probes |
| HyBR | CAATGACCGCTGTTATGCGG |  |
| FoSod5domBamHIf | CGCGGATCCGGATGACTCTCACGATGCTCC | 6×His-FoSod5 protein expression |
| FoSod5domHindIIIr | CCCAAGCTTTTAGGGACTAACAACGCTAGTCTC |  |
| qEF1αF | CATCGGCCACGTCGACTCT | Expression of *FoSOD5* by qRT-PCR |
| qEF1αR | AGAACCCAGGCGTACTTGAA |  |
| qFoSOD5F | GTCCAGTTCAAGGTCCAGTTC |  |
| qFoSOD5R | GGCGGTACAGTTGCCATTAT |  |
| FoSod5sgRNA1 | aagcTAATACGACTCACTATAGGCTTTGCCCAACACGGATTGTTTTAGAGCTAGAAATAGCAAG | CRISPR/Cas9-mediated endogenous gene tagging to generate four FoSod5 variants |
| gRNAR | AAAAGCACCGACTCGGTGCCACTTTTTCAAGTTGATAACGGACTAGCCTTATTTTAACTTGCTATTTCTAGCTCTAAAAC |  |
| FoSod5TF | CGGGCTCGGAGAAGTGTAAC |  |
| FoSod5TR | TCGGAGCTTCAGCCTGGATA |  |
| NA_FoSod5C1hitiF | agtgaattcgagctcggtacccgggGCACTCGTTTTGCTTGTTG |  |
| NA_FoSod5C1hitiR | cgcccttgctcacTGCGGCATCTTTGAAGAAAG |  |
| NA_FoSod5C1sGFPHYGBF | caaagatgccgcaGTGAGCAAGGGCGAGGAG |  |
| NA_FoSod5C2uphitiR | cgcccttgctcacTGCGGCATCTTTGAAGAAAG |  |
| NA_FoSod5C2midsGFPF | caaagatgccgcaGTGAGCAAGGGCGAGGAG |  |
| NA_FoSod5C2midsGFPR | ccgggttcaagttCTTGTACAGCTCGTCCATGC |  |
| NA_FoSod5C2downF | cgagctgtacaagAACTTGAACCCGGGTGTTATC |  |
| NA_FoSod5C2downR | ggcatgatggttgTCGACTGATTGCGCTACTC |  |
| NA_FoSod5C2HYGBF | cgcaatcagtcgaCAACCATCATGCCACCAATATATTAATG |  |
| NA_C4_1R | gtccttgtagtcCTTGTACAGCTCGTCCATGC |  |
| NA_C4_2F | cgagctgtacaagGACTACAAGGACGACGTGAGCAAGGGCGAGGAGC |  |
| NA_C4_2R | cgtcgattgcggcGTAAGCGCCAGCGCTCTTGTACAGCTCGTCCATG |  |
| NA_C4_3F | gctggcgcttacGCCGCAATCGACGGCAATGT |  |
| NA_C4_3R | gcatgatggttgTCGACTGATTGCGCTACTCAAC |  |
| NA_Psod5R | cgacgggatccatCTTGAGTGAGATGGTAAAGC |  |
| NA_lacZF | catctcactcaagATGGATCCCGTCGTTTTAC |  |
| NA_lacZR | tcattcaacccccTTATTTTTGACACCAGACCAAC |  |
| NA_Tsod5_HYGBF | gtgtcaaaaataaGGGGGTTGAATGATGAAACTG |  |
| Universal_NA_for HITI TR | aacagctatgaccatgattacgccaAGGGTTGCGAGGTCCAATG |  |


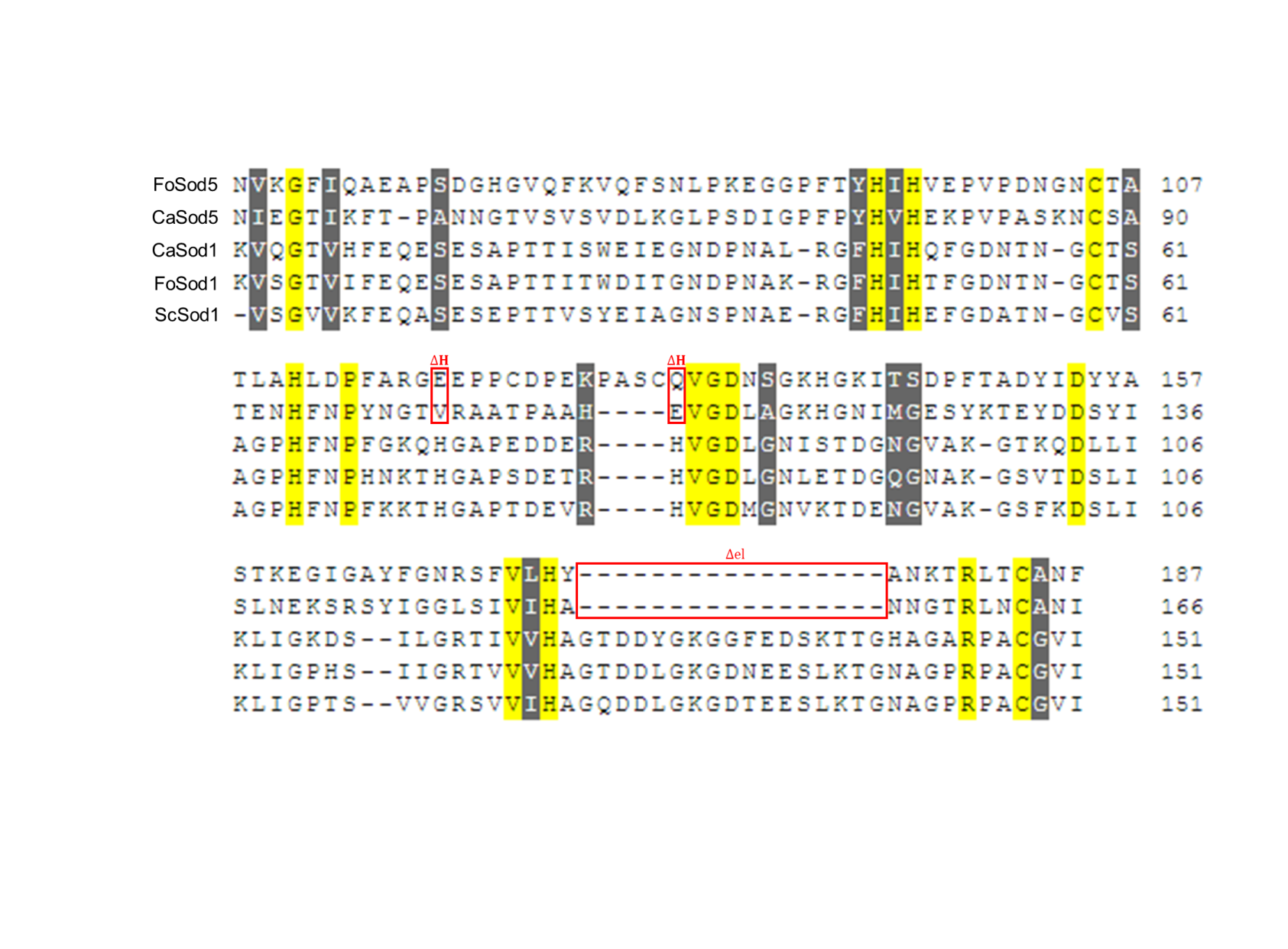


**Figure S1** Multiple amino acid sequence alignment of the SOD domain from FoSod5, CaSod5 (*C. albicans*), CaSod1, FoSod1, and ScSod1 (*S. cerevisiae*). The two histidine residues involved in zine binding that are absent in the *SOD5* orthologs are indicated by ∆𝐇, and the ∆el represents the missing Sod1 electrostatic loop, and provide evidence that FoSod5 is a Cu-only binding enzyme. R181 is a conserved amino acid site required for the activity of SODs.


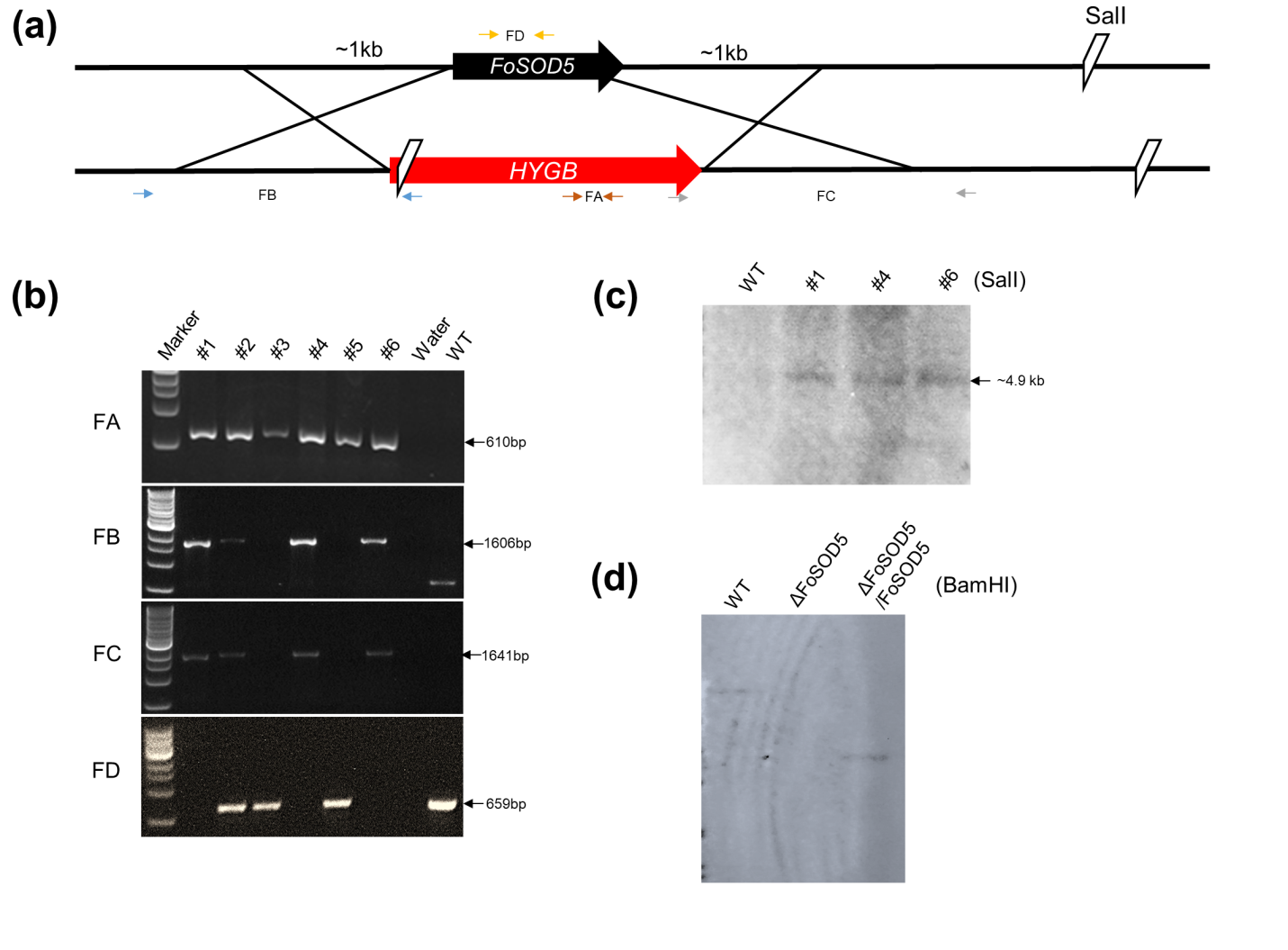


**Figure S2** *FoSOD5* gene disruption and complementation strategy. a) The ~1 kb upstream and ~1 kb downstream regions of *FoSOD5* were ligated flanking the hygromycin phosphotransferase (*hph*) cassette by overlapping PCR. b) Four pairs of primers were used to screen for the desired location of integration for the HPH cassette. FA: c) Southern hybridization to confirm a single integration of the HPH cassette occurred in the *F. oxysporum* genome. d) Southern blot to confirm a single *FoSOD5* gene cassette integrated into the *ΔFoSOD5* strain.


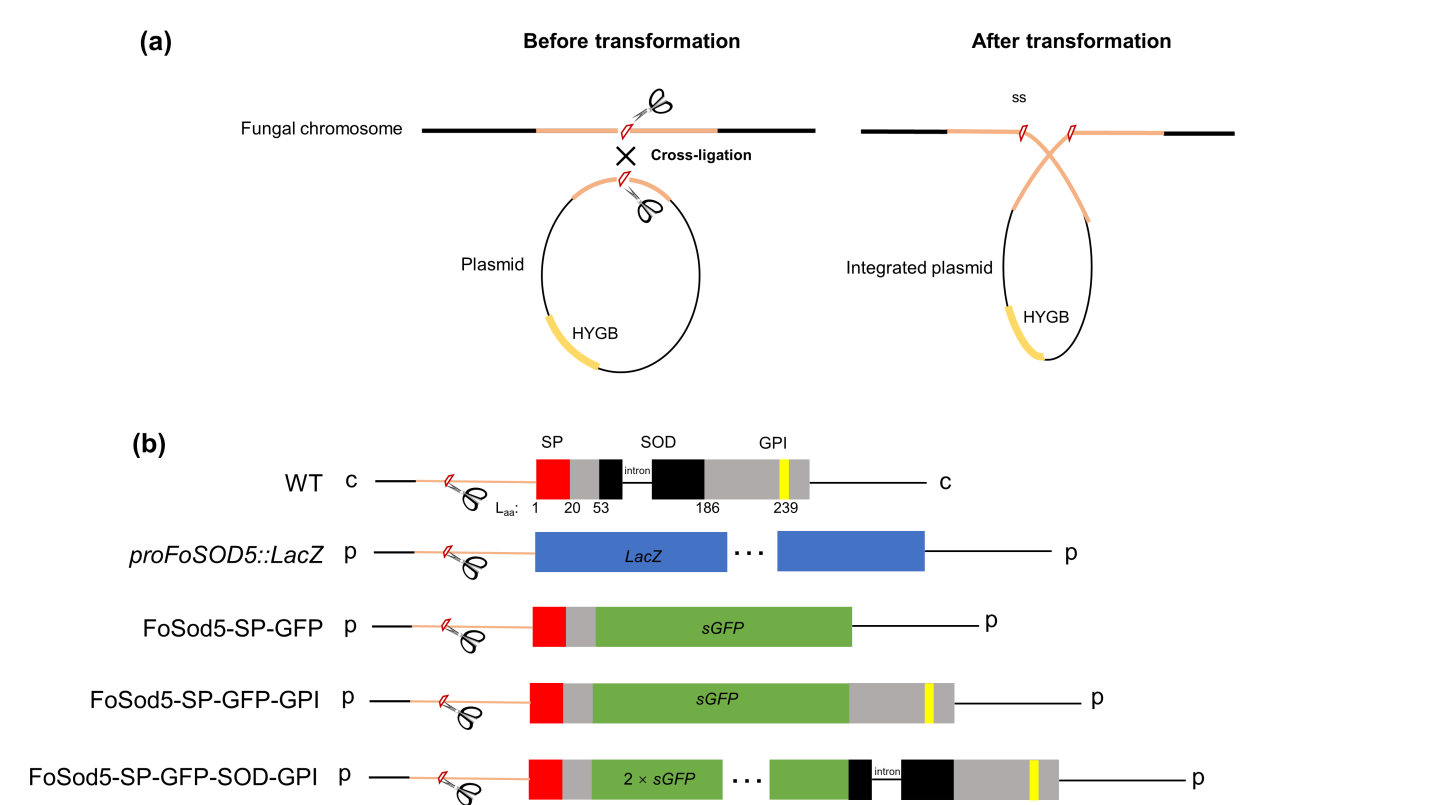


**Figure S3** CRISPR/Cas9 RNP-based transformant generation for FoSod5 GFP and LacZ variants. a) The homologous-independent targeted integration (HITI) strategy is illustrated and the desired transformant containing the donor plasmid integrated into the endogenous *FoSOD5* locus with the hygromycin cassette as the selective marker b) Schematic diagram of three different FoSod5 constructs. pro*FoSOD5::LacZ*, the LacZ reporter to monitor FoSOD5 expression; FoSod5-SP-GFP where sGFP replaced the SOD domain and GPI site; FoSOD5-SP-GFP-GPI where sGFP replaced the SOD domain; and FoSod5-SP-GFP-SOD-GPI inserting 2×*sGFP* between the secretion signal and the SOD domain.


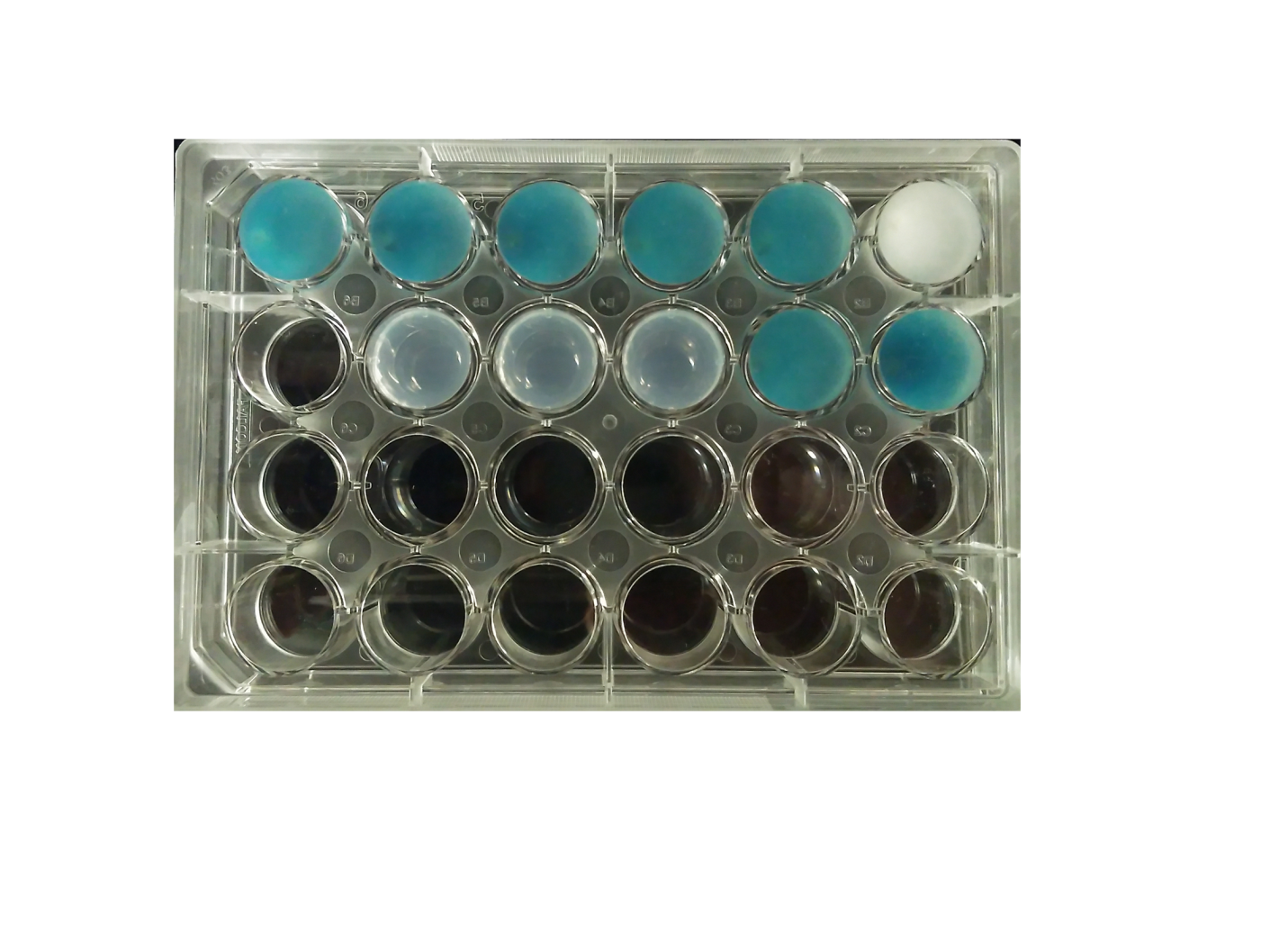


**Figure S4** The initial selection assay for generating the pro*FoSod5*::*LacZ* transformants. The minimum nutrient medium contained 150 μg/mL hygromycin and 200 ng/mL X-gal. Seven of the eight selected transformants had color changes, indicating the *lacZ* gene is expressed.


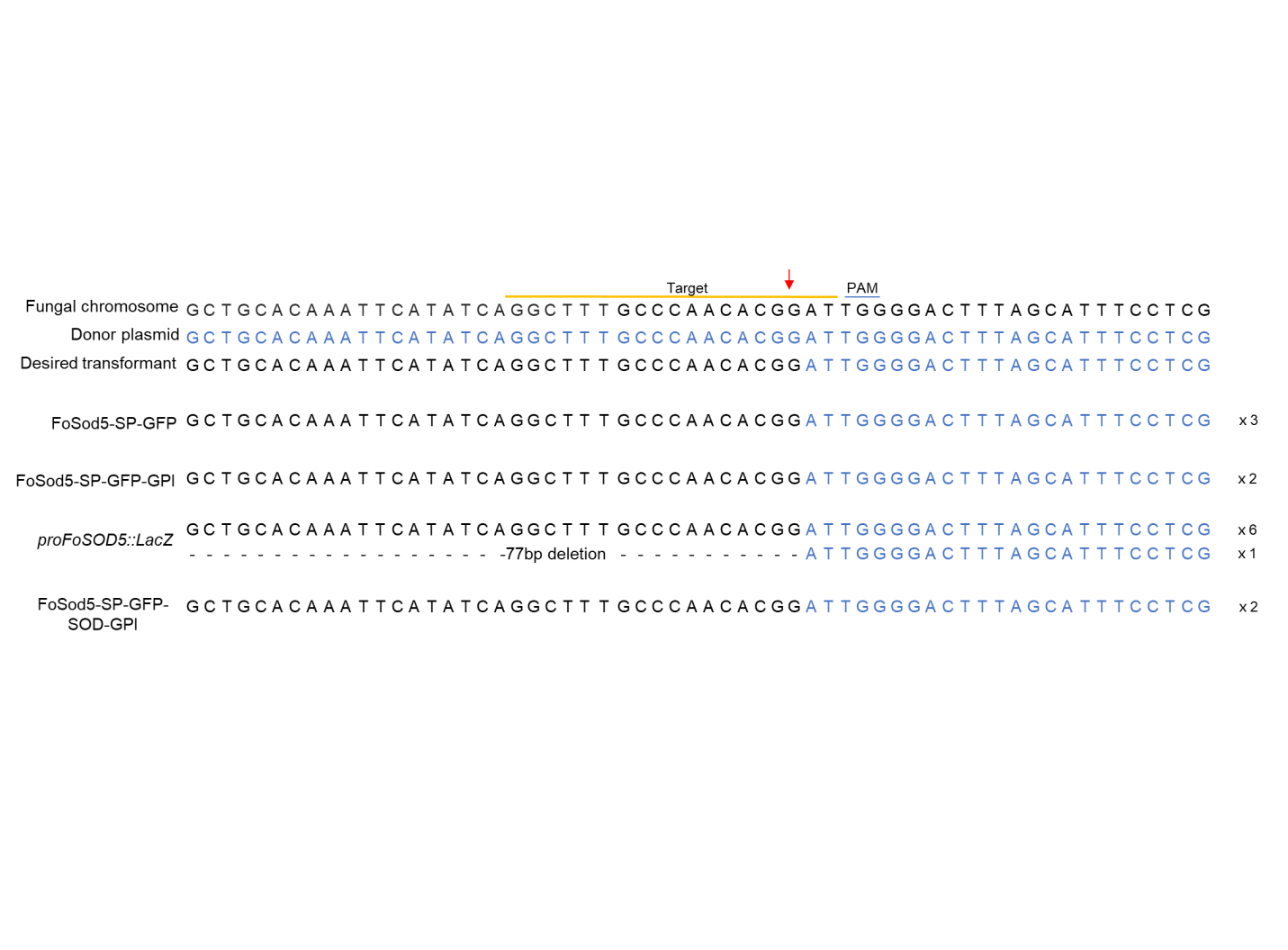


**Figure S5** The DNA sequences of the target region of FoSod5-SP-GFP, FoSod5-SP-GFP-GPI, *proFoSOD5::LacZ* and FoSod5-SP-GFP-SOD-GPI transformants. The red arrow represents the cleavage site of the Cas9 RNPs.


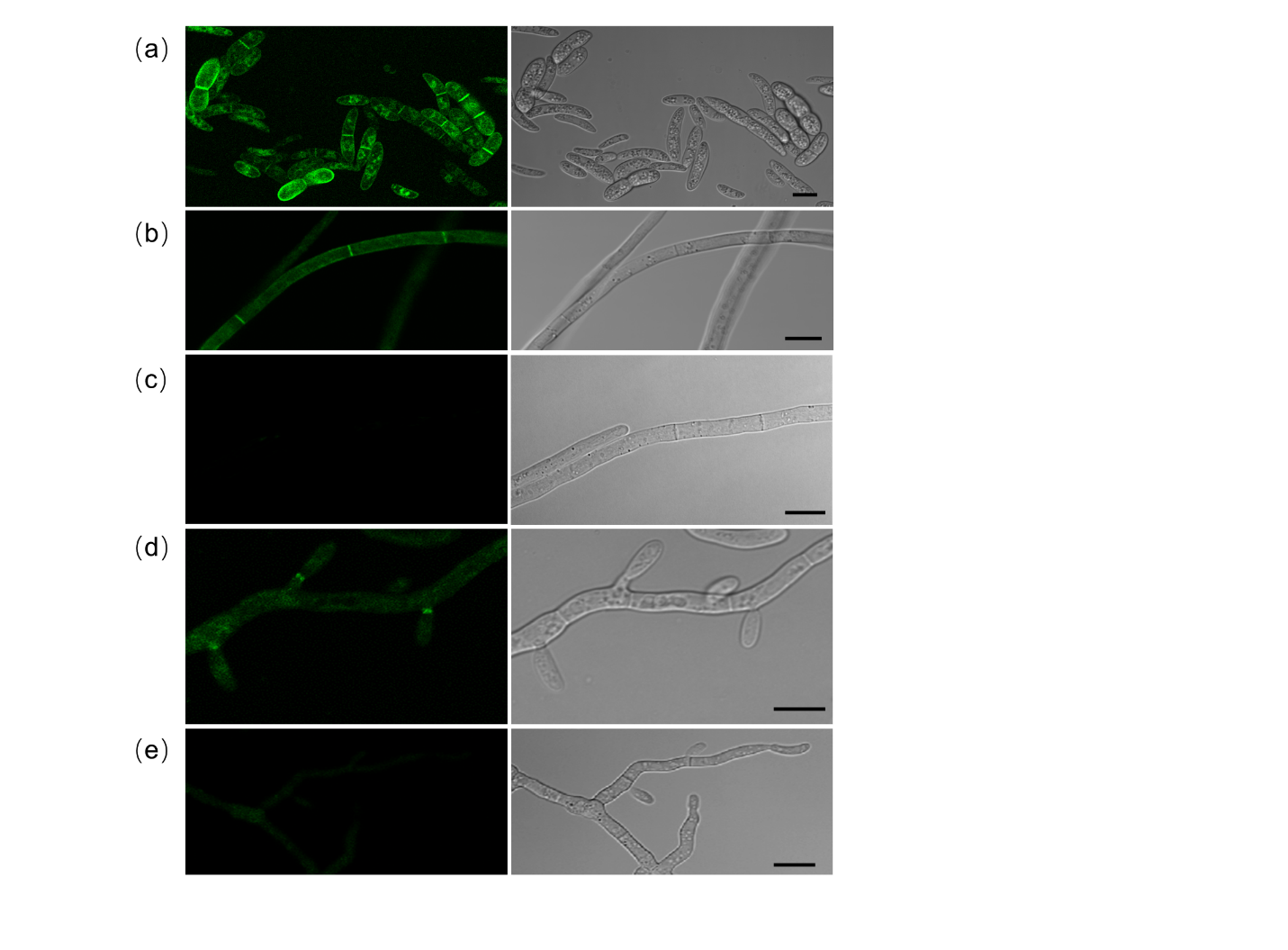


**Figure S6** GFP subcellular localization of the FoSod5-SP-GFP-SOD-GPI strain and the WT isolate under confocal microscopy. a,b) GFP subcellular localization of the FoSod5-SP-GFP-SOD-GPI strain in M-100 medium; c) and e) WT subcellular localization in M-100 medium and YG medium d) GFP subcellular localization of the FoSod5-SP-GFP-SOD-GPI strain in YG medium. Scale bars represent 10 μm.
